# Compliance and adherence in glaucoma management

**DOI:** 10.4103/0301-4738.73693

**Published:** 2011-01

**Authors:** Alan Robin, Davinder S Grover

**Affiliations:** 1 Department of Glaucoma, University of Maryland, 6115 Falls Road, Suite 333, Baltimore, MD, USA; 2 Department of Glaucoma, and Johns Hopkins University, 6115 Falls Road, Suite 333, Baltimore, MD, USA; 3 Glaucoma Associates of Texas, Dallas, TX, USA

**Keywords:** Adherence, compliance, glaucoma, glaucoma management, persistence

## Abstract

Glaucoma is a preventable cause of blindness if timely effective and successful treatment is provided. Patient adherence to the medication is a constant challenge that is now recognized as an essential component to treatment. Several studies have demonstrated that patients are more likely to be adherent to their medication if they understand the disease and the rationale for treatment and if their treatment regimen is simplified. Additionally, using eye drops has its own set of challenges that must be recognized and addressed at the clinical level. Although numerous socioeconomic factors are associated with poor adherence, these factors must be addressed at the societal level. Maximizing patient adherence to medication has the potential to reduce the number of surgical interventions required to treat glaucoma, prevent unnecessary vision loss, and save the overall healthcare system money in the long run.

Glaucoma, a chronic, progressive, and most often asymptomatic disease, is the second leading cause of blindness worldwide [World Health Organization (WHO)] but the leading cause of preventable blindness in multiple racial groups. In India, it is the leading cause of treatable non-reversible blindness. Risk factors for glaucoma, such as older age and higher intraocular pressure (IOP), are neither specific nor sensitive enough for mass screening[1] Additionally, although screening on a small scale may be of limited value, screening on a societal level is ineffective.[2] Although not “curable”, glaucoma is treatable and the primary objective of glaucoma therapy is to prevent progressive vision loss, disability, and blindness. The global burden of this disease disproportionately affects the developing world, and India is no exception. India has a population of over 1 billion people and has a huge burden of visual impairment and blindness.[3] Additionally, a large percentage of India’s population is rural where again a disproportionate percentage of glaucoma is found.[1] In fact, a population-based study in southern India demonstrated that glaucoma was the second leading cause of bilateral blindness, being responsible for 10% of cases detected in their study population.[4] The Aravind Comprehensive Eye Survey found that of the individuals diagnosed with glaucoma in the community, 50% had never had an eye examination and only 16% had ever visited an ophthalmologist. Therefore, not only are a large number of patients losing vision from glaucoma but also many of these patients are not accessing care.[4] An additional complicating factor to glaucoma treatment is that a large proportion of patients have been shown to have poor adherence. Several studies have demonstrated that roughly 50% of patients have been found not to be adherent to their medication over 75% of the time.[5] Although some workers have suggested that physician education can increase physicians’ appropriate style of communication with patients,[6] it may be extremely difficult to add more time to each patient interaction in a situation where the backload of cataract blind is so large. Additionally, there is a strong link between both adherence and health literacy in the developing world[7] and basic health literacy.

Therefore, multiple challenges exist for ophthalmologists in developing countries. The first challenge is ensuring that patients have access to medical and ophthalmic care. The next is to encourage patients to utilize this care. Once care is provided, the next challenge is ensuring that patients continue to adhere and utilize their treatment regimen.

We discuss the importance of compliance in chronic therapy, review the current literature on compliance and then discuss ways to incorporate compliance into one’s clinic practice in this review.

## Definition of Compliance and Adherence

Before discussing the impact of barriers to healthcare utilization and adherence with glaucoma management, it is essential to first define compliance and adherence.

Throughout the literature, compliance and adherence are often used interchangeably.[8] Traditionally, compliance has been defined as the extent to which patients’ behaviors correspond with physician’s recommendations.[9] Most healthcare professionals who are experts in this field prefer the term adherence because compliance connotes a degree of passivity on the patient’s part.[8] Some consider compliance as following the regimen on a daily basis, whereas persistence is for being compliant for longer duration. Another important term in this discussion is persistency, which is the total time for which the patient correctly takes the appropriate medication. Persistency is thought in part to represent the patient’s satisfaction with the agent’s tolerability as well as the physician’s satisfaction with the agent’s clinical efficacy.[9] However, this approach is most likely too simplistic. In reality, persistency also relies on additional factors such as cost, side effects, dosing frequency and many others.

## Why Do We Need Adherence in Chronic Therapy?

Although the correct diagnosis is an essential component in the management of this disease, the appropriate treatment is of equal, if not greater, importance. Patients with glaucoma most often require lifelong treatment and follow-up care to preserve vision. The current standards for glaucoma treatment range from topical medications and laser procedures to incisional surgery. Glaucoma is a preventable cause of blindness; however, numerous studies, in both developed and less developed countries, have demonstrated that access to eye care resources and adherence to treatment are still the major obstacles.[3,10] Once the diagnosis of glaucoma has been established, effective treatment is paramount. Treatment involves both rationale therapeutic interventions and patient adherence to the intervention. The therapeutic benefit from medication is maximized when administered correctly.

## Importance in Clinical Practice

Patient compliance to their medication regimen is essential for treating most chronic diseases; glaucoma is no exception. Poor adherence to medication regimens accounts for substantial worsening of disease and increased healthcare costs.[11,12] Diseases such as diabetes, hypertension, and glaucoma are most problematic because they are typically asymptomatic until the late stages. When patients are without symptoms, they may not realize the importance of daily adherence.[9] This is in contrast to diseases where patients are usually immediately symptomatic if they do not adhere to their medical regimen, such as seasonal allergies or pain medication. However, even in very symptomatic diseases requiring radical therapy such as mastectomy, adherence to tamoxifen diminishes to almost 50% at 2 years.[13]

During most clinical trials, physician think that the average rate of adherence is high, given the selection criteria for motivated patients and the ongoing monitoring and attention that the study patients receive. Despite the bias toward adherence, various clinical trials have reported average adherence rates of 43–78% among patients receiving treatment for chronic conditions.[8] Although there are no standard criteria for adherence rates in clinical trials, most clinical trials consider rates greater than 80% to be acceptable.

Prior studies have evaluated adherence either directly or indirectly.[5,14] Direct methods of monitoring involve either observing the patient take the medication or measuring concentration of the drug or metabolite in blood or urine. Indirect methods of assessing adherence include physician-estimated patient compliance, patient self-reporting, evaluating pharmacy refill rates, measuring the amount of medication in the bottle at each visit, utilizing electronic medication monitors, measuring clinical response, or using a patient completed medication diary. Each method has its advantages and disadvantages and no technique is without flaws. Although there is no consensus on the best method for measuring adherence, most of the studies have concluded that physicians are poor at predicting the degree of patient compliance and patients consistently overrepresent their degree of adherence.[8,14,15]

Additionally, trends in patient compliance are interesting. Several studies have demonstrated that patient adherence with medication improves in 5 days before and after their appointment with their physician.[5,8] This behavior pattern must be taken into account when treating patients who have progressive glaucomatous damage but are at a seemingly “safe” pressure. IOP control is a surrogate endpoint for successful therapy. If one considers both the effects of diurnal variation of therapy[16,17] and the effect of lack of adherence, except for the few days prior to the visit where IOP is measured, one might incorrectly believe that the IOP has always been adequately lowered, yet in reality, the patient had only been adhering for a few days during a 3-month interval.

Adherence to ophthalmic medications has a unique set of challenges compared to oral medications. Vrijens and coworkers[18] have described the various stages of adherence starting with acceptance, persistency, and the ability to “execute” or correctly administer a medication. Unless a patient has severe tremors, dementia, or dysphagia, the task of taking an oral medication is relatively simple and does not require observation or training by the treating physician. Although the concept of eye drop therapy is centuries old, little thought has been given to successful administration of eye drops. Eye drops are far more challenging to self-administer. Self-administering drops requires coordination, manual dexterity, eye hand coordination and good vision (all of which tend to decrease in aging glaucoma patients). Studies have also shown that adding a second medication and/or increasing the complexity of glaucoma therapy is associated with a statistically significant decrease in adherence.[19] Poor adherence is compounded if the drop is not appropriately placed on the eye. This issue is even more complex if we also consider other systemic medications a patient may be taking in addition to their glaucoma medications (New York Times/health/image/medication for complex diabetes/August 20, 2007). Various medications, with various routes of administration, may further complicate the issue. Often we only consider ophthalmic medications. However, many of our older patients are also on medications for diabetes, cholesterol, depression, systemic hypertension, osteoporosis, and hormonal replacement therapy, to name a few. The amount of administered medications may become staggering if we also consider various other homeopathies.

This dilemma creates a cycle of diminishing returns. The lack of proper adherence to a medication may lead to unnecessarily changing the treatment regimen or the addition of more medication (further compounding the problem) or perhaps an operation. Some authors have demonstrated that the more medication a patient is prescribed, the less likely the patient is to comply with the treatment regimen.[19]

Various factors have consistently been associated either positively or negatively with adherence. Patients knowledgeable about glaucoma, the disease course and consequences were consistently more compliant with their medication.[14,20] Other authors have found an association between the presence of depression and level of adherence.[5] There is an inverse relationship between number and frequency of dosage and patient adherence.[19,20] Interestingly, many studies did not detect an association between adherence and medication side effects.[5,20] When patients were questioned about barriers to adherence, the following reasons are often cited: forgetfulness (30%), other priorities (11%), lack of information (9%), emotional factors (7%) and 27% of individuals surveyed did not provide a reason.[8]

Physicians also play a major role in influencing their patients’ compliance. By prescribing complex therapeutic regimens, failing to explain the benefit and side effects of a medication adequately, not giving consideration to the patient’s lifestyle or the cost of the medications, and having a poor relationship with the patient – all have been shown to be associated with poor patient compliance.[5,8,14]

Fortunately, various strategies can be employed in an effort to improve patient adherence. Previous authors have demonstrated that educating the patient and family, simplifying the treatment regimen, involving family and friends, and customizing the treatment regimen to the patient’s lifestyle can improve patient adherence to their medication regimen.[5,9] Konstans and co-workers randomized subjects to education regarding adherence and attention placebo control, and have found a marked increase in adherence in newly diagnosed glaucoma patients.[21]

Patient adherence is extremely important for effective and successful treatment of their disease. Moreover, there is also a societal benefit to maximizing patient adherence. Poor adherence to medication has been shown to increase healthcare costs in the United States. According to Osterberg *et al*., of all medication related hospital admissions in the US, 33–69% are due to poor medication adherence, with a resultant cost of around $100 billion a year.[8]

Interestingly, no study to date has demonstrated an association between poor compliance and glaucomatous progression. This may in part be due to the fact that glaucoma is a slowly progressive disease; it can take three or more visual fields to accurately document perimetric progression and patient’s paper diaries of medication use may not be very accurate in assessing adherence.[22] However, numerous studies have demonstrated that treating ocular hypertension or glaucoma with ocular hypotensive agents delays the onset of primary open-angle glaucoma or progression of visual field loss.[23] Conceptually, based on these prior studies, one can argue that a certain degree of noncompliance with glaucoma treatment should be a risk factor for the progression of visual field loss.[19] If a once daily medication lasts only 24 hours, and if a patient misses one dose per week, the patient is missing more than 6 weeks of therapy per year.

Hand-in-hand with adherence to medication is the appropriate utilization of ophthalmic care with timely follow-up visits and obtaining access to care. Given the chronic and progressive nature of glaucoma, close and regular follow-up is essential. Patients depend upon their health infrastructure and resources in order to access routine and regular medical care. A recent study characterized some of the challenges associated with continuing glaucoma follow-up in southern India.[24] This study demonstrated that the most significant factors linked with poor follow-up were a lack of formal education and a belief that follow-up is less important if one uses glaucoma medications. Moreover, the most prevalent barriers to follow-up were the belief that there was no problem with one’s eyes and lack of escort to accompany the patient during their clinic visit. Interesting, but perhaps not surprising, is the fact that many of the factors associated with poor adherence to medication were found to be associated with poor follow-up to clinic visits.[25]

## Conclusion

Glaucoma is a preventable cause of blindness if effective and successful treatment can be provided at the appropriate time. As with many other fields of medicine, patient adherence to the medication is a constant challenge that is now recognized as an essential component of the treatment plan. Several studies have demonstrated that patients are more likely to be adherent to their medication if they understand the disease and the rationale for treatment and if their treatment regimen is simplified. Although there are numerous socioeconomic factors that have been associated with poor compliance, these factors must be addressed at the societal level as opposed to the clinical level. Maximizing patient adherence to both medical and surgical therapies, medication has the potential to reduce the number of surgical interventions required to treat glaucoma, prevent unnecessary vision loss, and save the overall healthcare system money in the long run. When considering adherence to topical ophthalmic medications, one must also not forget to include the problems of getting a drop into the eye.
